# Variation in Shear Force, Cooking Loss, pH, and Sarcomere Length of Eight Different Muscles Collected from Australian Rangeland Goats

**DOI:** 10.3390/foods15173126

**Published:** 2026-09-03

**Authors:** Benjamin W. B. Holman

**Affiliations:** Gulbali Institute, Charles Strut University, Wagga Wagga, NSW 2768, Australia; bholman@csu.edu.au

**Keywords:** chevon, muscle-specific variation, cut comparison, ultimate pH, tenderness, Warner−Bratzler shear force, water-holding capacity, principal component analysis (PCA)

## Abstract

Goat meat is often underutilised, partly due to inconsistent eating quality and limited information on variation between individual cuts or muscles. This study established a quality baseline for eight muscles from intact male Australian Rangeland goats by comparing cooking loss, shear force, ultimate pH and sarcomere length. The longissimus thoracis et lumborum, psoas major, semitendinosus, semimembranosus, adductor femoris, triceps brachii, rectus femoris and vastus lateralis muscles were sampled from 25 carcasses. Analysis demonstrated significant muscle effects for all quality traits (*p* < 0.05). Shear force ranged from 29.2 N in vastus lateralis to 50.2 N in semitendinosus, indicating substantial variation in tenderness. Sarcomere length varied from 1.50 to 1.99 µm, while cooking loss ranged from 12.5 to 19.4%. Ultimate pH was consistently high (6.14–6.33), reflecting the susceptibility of Australian Rangeland goats to this outcome. Exploratory correlation analysis demonstrated strong positive relationships and substantial concordance for ultimate pH across multiple muscles, indicating that it was highly consistent throughout the carcass. Principal component analysis found ultimate pH contributed most strongly to chevon quality variation, accounting for the largest proportion of total variation (30.5%), followed by cooking loss (17.2%) and tenderness-related traits (11.8%). These findings provide a foundational benchmark for Australian Rangeland goats and support targeted muscle utilisation, value-adding processing and the development of cuts-based marketing strategies to improve product consistency and consumer acceptance.

## 1. Introduction

Goat meat (chevon) is a popular lean red meat, but in Western markets, consumers often consider chevon as inferior to other red meats. This could be the result of chevon being less tender and juicy than beef and sheep meat, a quality difference that is likely due to its unique physicochemical properties, such as a lean carcass composition and the distribution of connective tissue [1,2]. Meat quality research has generally focused on a limited number of muscles, most notably the longissimus thoracis et lumborum (LTL) and semimembranosus (SM). A similar pattern is apparent for chevon, meaning there remains the potential to find more appropriate uses for specific muscles and to identify specific market needs. Effectively a ‘quality baseline’ is needed to better match different chevon muscles to market and consumer expectations. Research has already established benchmarks for different muscles collected across beef and sheep carcasses [3,4,5,6,7]. A quality benchmark is currently unavailable for different muscles collected from Australian Rangeland goats. Australian Rangeland goats are the primary source of animals for goat meat processing in Australia and are currently underutilised, being processed as whole carcasses or as frozen six-way cuts [8]. It is therefore important to address this paucity to support a cuts-based market and improve appeal to domestic consumers.

Tenderness is a critical determinant of eating quality and often objectively measured as shear force, being the resistance of a muscle to cutting [9]. Chevon often fails to achieve the high degree of tenderness achieved by other red meats, partly because goats lack the subcutaneous fat coverage necessary to protect carcasses from cold shortening during the chilling process [1]. Cold shortening leads to permanent muscle contraction, directly impacting the sarcomere length—being the distance between the functional units of muscle fibres [10]. Shorter sarcomere lengths are typically associated with tougher meat [10,11]. Furthermore, goats are widely considered temperamental animals, susceptible to stress and the rapid depletion of muscle glycogen reserves immediately prior to slaughter [8,12]. This insufficient glycogen reserve restricts post-mortem glycolysis and the accumulation of lactic acid and ADP, resulting in a high ultimate pH [13]. Meat with a high ultimate pH is characterised as dark, firm, and dry (DFD)—offering a product with poor shelf-life and consumer appeal [14]. In addition to ultimate pH, cooking loss is a key indicator of meat water-holding capacity and juiciness and refers to the amount of fluid lost during the cooking process. Collectively, these objective parameters provide valuable insights into meat quality and should be included into any ‘quality’ baseline comparison between different muscles.

Based on these examples and the literature, the research question posed is to what extent do instrumental measures of tenderness vary among the muscles from the same Australian Rangeland goat carcass? In response, this study aimed to compare the shear force, cooking loss, ultimate pH and sarcomere length of eight specific muscles collected from Australian Rangeland goats. By establishing these baseline parameters, this study will help improve product consistency and competitiveness.

## 2. Materials and Methods

### 2.1. Carcass and Sampling

A total of 25 intact male (billy) Australian Rangeland goat carcasses were randomly selected from the boning room of a commercial Australian abattoir (Nathalia, AUS). Prior to slaughter, the goats had been held in overnight lairage with ad libitum access to drinking water. The goats were slaughtered as a single group following the standard industry practices of head-only electrical stunning, exsanguination via a ventrolateral neck cut, then evisceration and dressing according to market specifications for skin-off carcasses. Hot standard carcass weights were recorded at ~35 min post-mortem. Carcass fatness was measured at the GR site, defined as the tissue depth over the 12th rib at a distance of 11 cm from the carcass midline, using a specialised GR knife. Carcasses were chilled overnight (2.4 ± 0.8 °C) before being transported under refrigerated conditions to Charles Sturt University Meat Laboratory (Wagga Wagga, AUS). Upon arrival, carcasses were maintained at 1.3 ± 0.6 °C until fabrication at 5 days post-mortem. Specifically, from the left side of each carcass, eight distinct muscles were excised, trimmed of external fat and connective tissue, and vacuum packaged. The muscles collected were the LTL, psoas major (PM), semitendinosus (ST), SM, adductor femoris (AD), triceps brachii (TB), rectus femoris (RF) and vastus lateralis (VL). These were then frozen at −20 °C until subsequent analysis, completed within 3 months of collection.

### 2.2. Cooking Loss and Shear Force

Frozen samples were cooked from frozen in 5 batches, with carcass allocation to batch randomised, although batches were balanced by muscle within carcass. The samples were cooked to an internal temperature of 71 °C using a water bath (TWBC-24-TU3, Thermoline Scientific, Sydney, Australia). Temperatures were verified using an infrared HACCP thermometer with probe attachment (model 8838, AZ Instrument Corp., Taipei, TAI, China) and the samples were then submerged in an ice slurry for 30 min to halt the cooking process. The percentage change in sample weight following cooking was calculated as cooking loss. Cooked samples were placed into resealable plastic bags and refrigerated until their temperatures equilibrated to 3–4 °C. Four to six cuboidal strips, with a 1 cm^2^ cross-sectional area, were prepared from each sample parallel to the muscle fibre orientation. Where fewer than six strips were available, repeated measurements were conducted on the available strips until six valid readings were obtained. The peak force (Newtons) required to sever these strips was measured using a texture analyser (XT-Plus 100C, Stable Micro Systems, Godalming, UK) fitted with a Warner−Bratzler shear force blade and set to a 200 mm/min crosshead speed. The cutting line was positioned to be perpendicular to the muscle fibre orientation and to avoid fatty deposits and connective tissues. As per Holman et al. [15], the average of 6 repeat measures was recorded as the shear force.

### 2.3. Ultimate pH

Frozen samples (1 g) were homogenised with 6 mL of buffer (150 mM KCl, 5 mM iodoacetate) and then incubated in a circulating water bath (TWBC-24-TU3, Thermoline Scientific, AUS) until temperatures equilibrated to 20 °C. pH was measured using a pH meter fitted with a spear-type gel electrode (WP-80 and IJ-44 Iodone respectively, TPS, Brisbane, Australia) calibrated using pH 4.01 and 7.00 standards at 20 °C. As per De Brito et al. [16], the average of 2 repeat measures was recorded as ultimate pH (PHU).

### 2.4. Sarcomere Length

The average sarcomere length (µm) was calculated as per Bouton et al. [17], based on the results from 5 thin slices of <1 mm thickness, removed from different locations across each frozen sample parallel to the muscle fibre orientation. The slices were gently pressed between glass slides and then analysed using a laser light diffraction unit.

### 2.5. Statistical Analysis

Data were analysed using Stata/SE (v18.0 StataCorp LLC, College Station, TX, USA), having first been checked for imputation errors and outliers. Linear mixed models using restricted maximum likelihood (REML) were fitted with the fixed effect of muscle and random effect of carcass (block). Cooking batch was included as a covariate for the analysis of cooking loss and shear force data [18]. Pre-cook sample block weight was fitted as an additional random effect for the analysis of cooking loss data. Statistical differences between fixed effects were identified using Bonferroni’s multiple comparison test at *p* < 0.05.

Pairwise relationships between muscle and quality trait combinations were evaluated using Pearson’s correlation coefficients and Lin’s concordance correlation coefficient. The former was used to quantify the strength of linear associations between variables [19], whereas the latter was used to assess absolute agreement by quantifying the extent to which observations deviated [20]. Concordance was further partitioned into precision and accuracy, quantified by the bias correction factor to account for scale and location shifts from equality. Concordance analyses were restricted to within-quality-trait comparisons across muscles, to evaluate whether one muscle could serve as a proxy for another. Given the nature of correlation analyses and the large number of pairwise comparisons performed, significant relationships should be interpreted as indicative of collinearity rather than causal. Consequently, multivariate analysis was performed to further scrutinise the results from these analyses.

Principal component analysis (PCA) was performed on the standardised (*z*-score transformed) dataset comprising 32 variables (8 muscles × 4 quality traits). Eigenvalues were extracted from the correlation matrix and principal components were retained based on the Kaiser–Guttman criterion (eigenvalue of ≥1.0) and a visual assessment of the scree plot [21]. The retained component loadings were subjected to orthogonal varimax rotation and examined to identify latent dimensions and co-varying quality traits among muscle types.

## 3. Results

The hot carcass weight and GR tissue depth for the Australian Rangeland goats used in this study were 15.8 ± 5.2 kg and 2.0 ± 1.3 mm, respectively.

### 3.1. Muscle Effects

There were significant differences observed between muscles for each of the quality traits evaluated (*p* < 0.001; Table 1). Cooking loss ranged between 12.5 and 19.4%, with LTL having the highest cooking losses, except when compared to SM, which had cooking losses consistent with all other muscles. Sarcomere length ranged between 1.50 and 1.99 µm. The longest sarcomeres were found in AD, followed by TB, whereas the SM and ST had the shortest sarcomere lengths (1.50 and 1.52 µm, respectively). Shear force was highest in the ST (50.2 N) and lowest in the PM (32.7 N), RF (32.8 N) and TB (33.7 N). Ultimate pH also varied significantly between muscles, ranging from 6.14 and 6.33. The SM had the lowest ultimate pH, whereas the VL, TB, RF and PM had the highest (6.28–6.33).

### 3.2. Correlations

Significant correlations were identified between quality trait and muscle combinations (*p* < 0.05; Table 2). These included 27 positive correlations and 11 negative correlations, meaning that 38 of the 496 possible pairwise correlations (7.7%) were statistically significant. Most involved ultimate pH, which was found to have strong positive correlations between AD and SM (*r* = 0.931), ST and TB (*r* = 0.899), TB and VL (*r* = 0.888), and PM and TB (*r* = 0.882). Corresponding Lin’s concordance correlation coefficients ranged from 0.646 to 0.883, while bias correction factors were consistently high (*Cb* = 0.830 to 0.997). This indicates substantial agreement and little systematic bias between ultimate pH measurements across the muscles of Australian Rangeland goats.

Moderate positive correlations were found between SM sarcomere length and TB shear force (*r* = 0.795), AD and LTL shear force (*r* = 0.614), ST and TB shear force (*r* = 0.467), and cooking loss and shear force for SM (*r* = 0.447) and RF (*r* = 0.415). In contrast, ultimate pH was negatively correlated with shear force (*r* = −0.614 to −0.709) and sarcomere length (*r* = −0.636 to −0.712). The strongest negative relationship was found between AD ultimate pH and TB shear force (*r* = −0.709), and RF ultimate pH and SM sarcomere length (*r* = −0.712).

### 3.3. Principal Component Analysis

**Figure 1:**
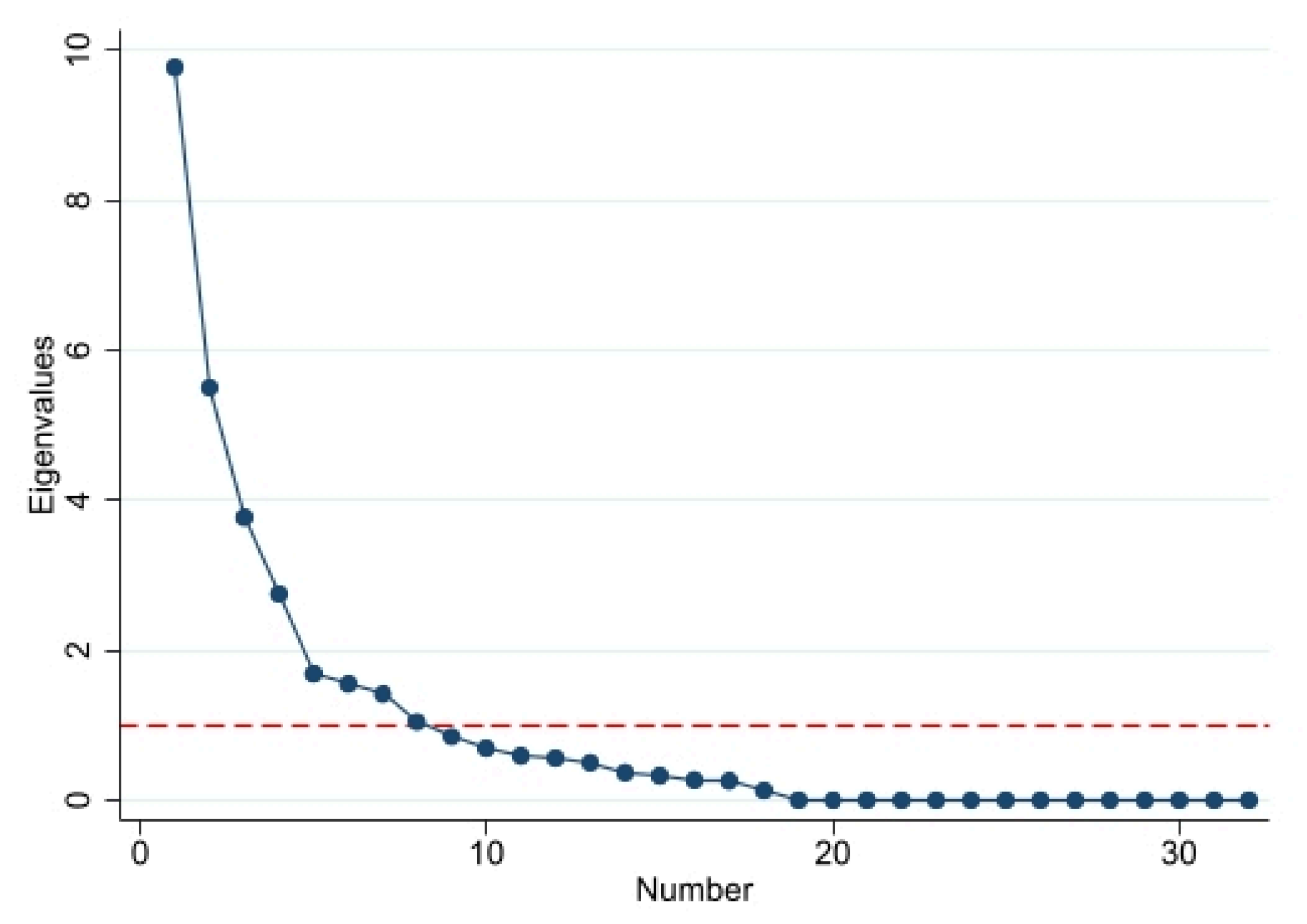
A scree plot of eigenvalues from the principal component analysis evaluating the quality traits of eight muscles from Australian Rangeland goats. The dashed red line indicates the Kaiser−Guttman criterion (Eigenvalue ≥ 1.0).

The scree plot (Figure 1) shows that the first four principal components had eigenvalues of >1.0 and collectively explained 68.1% of the total variance in quality traits across muscles. The first three principal components explained 59.5% of the total variance, with principal components 1, 2 and 3 explaining 30.5%, 17.2% and 11.8% respectively. All eight principal components collectively explained 85.9% of the total variance (Table 3).

**Figure 2:**
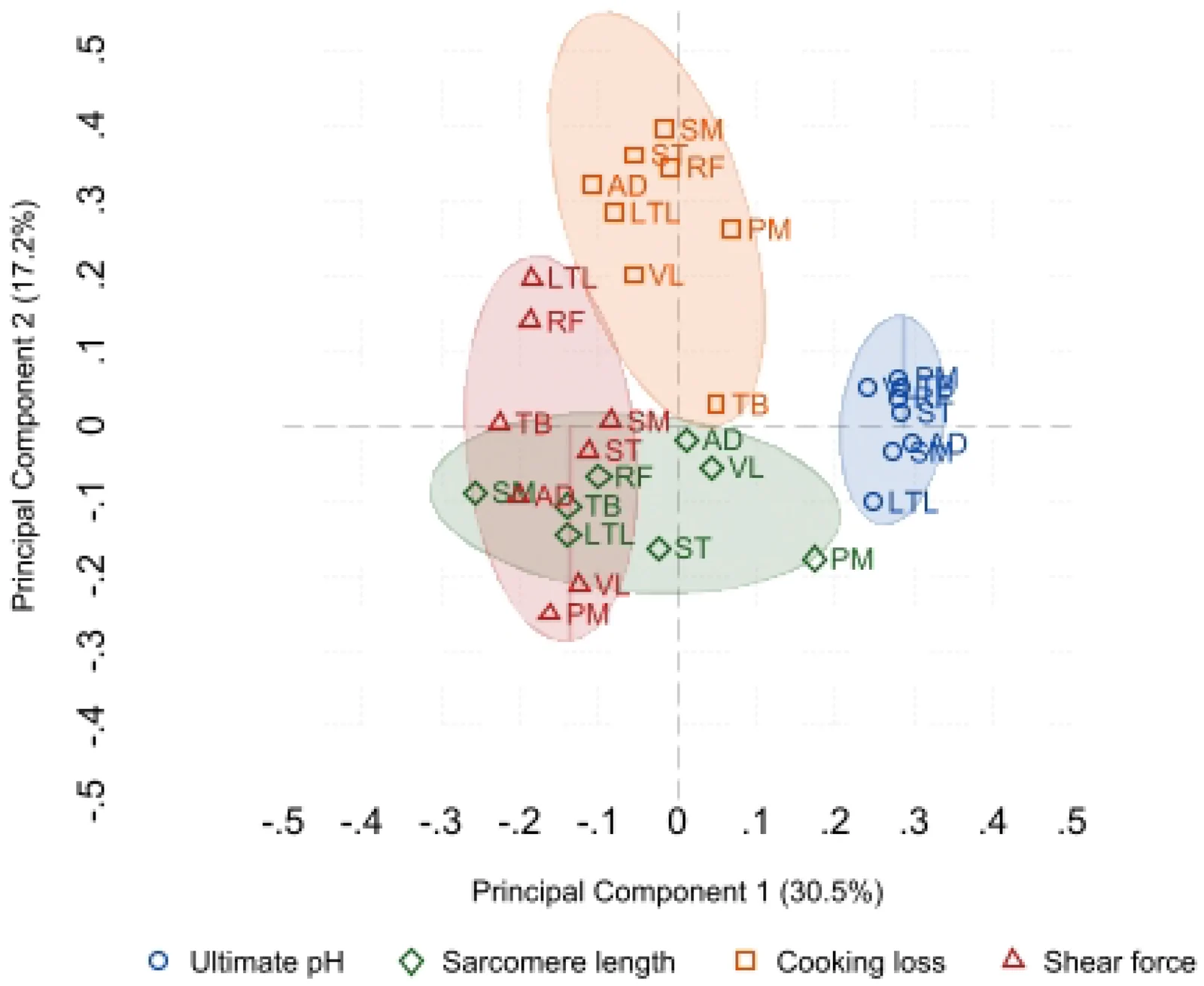
This principal component analysis biplot showing the distribution and relationship between the quality traits of eight muscles from Australian Rangeland goats. Principal components 1 and 2 account for 30.5% and 17.2% of total variance, respectively. Other abbreviations include the following: AD—adductor femoris; LTL—longissimus thoracis et lumborum; PM—psoas major; RF—rectus femoris; SM—semimembranosus; ST—semitendinosus; TB—triceps brachii; VL—vastus lateralis.

Principal component 1 was primarily associated with ultimate pH, having consistently positive loadings across all muscles (0.24–0.30), indicating that pH was the major contributor to overall variation in chevon quality traits. Principal component 2 was primarily associated with cooking loss, with the largest positive loadings observed for SM (0.39), ST (0.36), RF (0.34), AD (0.32) and LTL (0.28), suggesting that variation in water-holding capacity represented the second most important source of variation. Principal component 3 was primarily associated with tenderness-related traits, with positive loadings for shear force in AD (0.29), SM (0.32) and ST (0.29), together with negative loadings for sarcomere length in AD (−0.38) and VL (−0.27), reflecting the inverse relationship between muscle structure and tenderness. The remaining principal components explained between 3.3 and 8.6% of the variance each and captured more muscle-specific variation. Notable loadings included RF sarcomere length on PC5 (0.52), TB cooking loss on PC8 (0.55), LTL sarcomere length on PC7 (0.41) and ST shear force on PC6 (−0.41). The PCA biplot (Figure 2) further demonstrated that ultimate pH, cooking loss and tenderness-related traits contributed most strongly to variation in meat quality among the eight muscles examined.

## 4. Discussion

Significant muscle effects were observed for cooking loss, sarcomere length, shear force and ultimate pH, demonstrating that muscle selection is a major source of variation in Australian Rangeland chevon quality. Confidence in the representativeness of the sampled cohort was gained from comparison to previous studies of goat LTL quality traits [1,5,13,22,23,24], noting the few studies available that have explored muscle-specific differences. The exceptions were Dieters, Meale, Quigley and Hoffman [22], whose comparison of the LTL and biceps femoris (BF) of 52 feedlot-finished Australian Rangeland goats found similar muscle-specific effects on shear force, colour, fat content and drip loss; and van Wyk et al. [25], whose comparison of six muscles, sampled from 19 Indigenous Veld and 18 Boer goat carcasses, found similar quality trait variation between the SM, BF and ST to the LTL. Comparable muscle-specific differences have also been reported for other livestock species. Ithurralde, Bianchi, Feed, Nan, Ballesteros, Garibotto and Bielli [7], for example, compared 15 muscles from lamb carcasses and found variation in sarcomere length, shear force, and ultimate pH. Tschirhart-Hoelscher, Baird, King, McKenna and Savell [6] performed a comparison of 18 lamb muscles and found variation in shear force, sarcomere length, pH and water-holding capacities. Jeremiah, Gibson, Aalhus and Dugan [4] compared 33 muscles from beef steers and found variation in consumer sensory panel scores of tenderness and juiciness. Belew, Brooks, McKenna and Savell [3] compared 40 muscles from beef cattle and found variation in shear force. Such variation has been attributed to intrinsic differences in muscle fibre type and density, connective tissue, intramuscular fat deposition and the extent of post-mortem proteolysis [24,26].

Variation in shear force reflected the functionality of individual muscles. The PM, RF, TB and VL exhibited the lowest shear force values, whereas the locomotory ST and SM were consistently tougher. These differences are consistent with the greater connective tissue content and increased collagen cross-linking associated with muscles subjected to sustained locomotor activity [26,27,28]. Although consumer tenderness thresholds for chevon have not been established, comparison with lamb shear force thresholds [29] suggests that the PM and VL are suitably tender for premium fresh meat applications, whereas the ST and SM may be better suited to slow-cooking or value-added processing. All muscles in the current study were aged for only 5 days post-mortem. Extending the ageing period would be expected to promote further proteolytic tenderisation, with ~14 days proposed as necessary to maximise tenderness in lamb meat [30]. Consequently, longer ageing periods may reduce the differences in shear force observed between muscles or be targeted for inherently tougher cuts to improve their suitability for fresh retail markets [13]. Chaosap, Sitthigripong, Sivapirunthep, Pungsuk, Adeyemi and Sazili [26] have found that calpain and calpastatin concentrations vary between goat muscles, selected from Boer crossbred bucks, supporting muscle-specific ageing interventions to enhance tenderness. Although shear force measurements are widely used within meat science [29], consumer sensory responses were not evaluated in the current study. Therefore, classifications of muscles as premium or lower-value cuts should be considered provisional until validated through consumer sensory assessment, also being dependent on cooking methods, demography and culinary experience [31].

Differences in sarcomere length supported the observed muscle-specific trends in shear force, with the SM and ST exhibiting the shortest sarcomeres. Short sarcomeres are indicative of cold shortening [10], a recognised risk in lean goat carcasses, because limited subcutaneous fat permits rapid post-mortem chilling [5,32]. As electrical stimulation was not applied in the present study, cold shortening may have contributed to the reduced sarcomere lengths observed in these muscles. Nonetheless, the shorter sarcomeres observed in the SM and ST are consistent with their greater shear force values, supporting the contributions of sarcomere shortening to muscle-specific differences in tenderness. However, tenderness is a multifactorial trait influenced by not only sarcomere length but also connective tissue, collagen solubility and the extent of post-mortem proteolysis [5,28,33].

Cooking loss differed significantly among muscles, indicating differences in water-holding capacity [34]. Notably, the LTL exhibited relatively high cooking loss despite only intermediate shear force values, demonstrating that tenderness and juiciness represent distinct quality attributes governed by different structural and biochemical properties of muscle [34,35]. These findings suggest that muscle-specific differences in water-holding capacity should also be considered when selecting cuts for fresh retail markets or specific cooking applications.

The uniformly elevated pH values suggest that variation attributable to differences in glycogen depletion may have contributed substantially to the overall quality variation observed among carcasses. Consequently, the relative importance of muscle-specific effects reported herein should be interpreted in the context of a cohort exhibiting widespread DFD characteristics [14]. Ultimate pH is primarily determined by muscle glycogen concentration at slaughter, with reduced glycogen availability limiting post-mortem lactic acid production and resulting in elevated pH [36]. Australian Rangeland goats are particularly susceptible to pre-slaughter stress because of their extensive production systems, frequent mustering and temperament, all of which can deplete glycogen reserves before slaughter [8,12,23]. These findings suggest that interventions that minimise pre-slaughter stress would likely have a greater influence on overall consumer acceptance of chevon quality, compared to a muscle-specific or cuts-based marketing strategy. In practice, this suggests that further investigation is necessary to determine whether similar rankings among muscles would be observed in goats with conventional ultimate pH values (<5.8).

The observed correlations and concordance coefficients indicate that ultimate pH measurements were relatively consistent among muscles within this cohort. However, before completing any multiple exploratory comparisons, these relationships should be interpreted cautiously and validated independently before any muscle is proposed as a proxy for whole-carcass pH status. Nonetheless, these results do seem to show a systemic depletion of glycogen, demonstrating that pH was highly consistent throughout the carcass. Consequently, pH measurement in a single muscle may provide an indicative estimate of whole-carcass pH status and warrants further validation under commercial grading conditions. If appropriate, this finding supports the use of muscles with lower commercial value for routine quality assessment while reducing the risk of damaging premium cuts via direct measurement. Similar relationships have been reported in lamb, where ST pH has been shown to reflect LTL pH, allowing assessment of carcass pH status without compromising higher-value muscles [37]. That said, multifactorial analysis was required as correlation is not indicative of causation, only identifying linear relations and, unless appropriately weighted, cannot differentiate between true associations and chance [38]. Principal component analysis demonstrated that ultimate pH was the largest contributor to variation among the quality traits measured, accounting for 30.5% of the total variance, whereas cooking loss and tenderness-related traits explained additional independent sources of variation. The strong cross-loading of shear force and sarcomere length within principal component 3 further supports the biological relationship between myofibrillar shortening and tenderness. Previous research has reported the same relationship, albeit in other livestock species [39,40]. These results emphasise that chevon quality is determined by multiple biological mechanisms and reinforce the importance of assessing several complementary quality traits rather than relying on any single measurement [29].

## 5. Conclusions

This study demonstrates substantial variation in cooking loss, sarcomere length, shear force and ultimate pH among eight muscles from Australian Rangeland goats, confirming that muscle selection is an important determinant of instrumental meat quality. The VL displayed the most favourable overall combination of objective quality traits, while the PM, RF and TB were also among the most tender muscles. In contrast, the ST and SM exhibited shorter sarcomeres and greater shear force values, suggesting lower tenderness potential and greater suitability for value-added processing or slow-cooking applications. Despite these muscle-specific differences, ultimate pH was uniformly high across all muscles and represented the largest source of variation within the dataset, highlighting the importance of pre-slaughter management and glycogen preservation in Australian Rangeland goats. The strong concordance observed for pH among muscles further suggests that carcass pH status may be estimated from a single muscle, although additional validation is required, particularly in carcasses with both normal and high pH.

Collectively, these findings provide the first objective quality baseline for multiple muscles from Australian Rangeland goats and support the development of cuts-based utilisation strategies. However, the results should be interpreted in the context of single cohort of 25 intact male Australian Rangeland goats processed under one commercial production system. Consequently, the findings may not fully represent the variation associated with sex, age, season, nutrition, genetics or alternative processing conditions. Furthermore, quality assessment was limited to instrumental measurements and did not include sensory evaluation, fibre type, collagen profile, colour stability, retail shelf-life or drip loss measurements. Future research should include broader commercial populations and directly relate muscle-specific quality traits to consumer eating quality outcomes.

## Figures and Tables

**Table 1:** Predicted means, standard error (SEM) and level of significance for quality traits of eight muscles from Australian Rangeland goats ^1^.

Traits	Muscle								SEM	*p*-Value
	AD	LTL	PM	RF	SM	ST	TB	VL		
Cooking loss, %	12.5 ^a^	19.4 ^b^	15.6 ^a^	13.0 ^a^	15.6 ^ab^	13.0 ^a^	15.5 ^a^	12.5 ^a^	1.2	<0.001
Sarcomere length, µm	1.99 ^e^	1.57 ^ab^	1.65 ^abc^	1.71 ^bcd^	1.50 ^a^	1.52 ^a^	1.86 ^de^	1.78 ^cd^	0.06	<0.001
Shear force, N	38.4 ^bc^	42.4 ^cd^	32.7 ^ab^	32.8 ^ab^	45.5 ^cd^	50.2 ^d^	33.7 ^ab^	29.2 ^a^	2.5	<0.001
Ultimate pH	6.25 ^ab^	6.23 ^ab^	6.28 ^b^	6.31 ^b^	6.14 ^a^	6.22 ^ab^	6.31 ^b^	6.33 ^b^	0.04	<0.001

^1^ Different superscript within a row indicate significant differences between muscles. Other abbreviations include the following: AD—adductor femoris; LTL—longissimus thoracis et lumborum; PM—psoas major; RF—rectus femoris; SM—semimembranosus; ST—semitendinosus; TB—triceps brachii; VL—vastus lateralis.

**Table 2:** Significant (*p* < 0.05) Pearson’s correlation coefficients (*r*) for quality traits of eight muscles from Australian Rangeland goats. Lin’s concordance correlation coefficients (*pc*) and bias correction factors (*Cb*) were included for same-quality-trait comparisons ^1^.

Pair One		Pair Two		*r*	*pc*	*Cb*
Muscle	Trait	Muscle	Trait			
AD	Ultimate pH	SM	Ultimate pH	0.931	0.883	0.949
ST	Ultimate pH	TB	Ultimate pH	0.899	0.863	0.960
TB	Ultimate pH	VL	Ultimate pH	0.888	0.871	0.981
LTL	Ultimate pH	SM	Ultimate pH	0.882	0.842	0.954
PM	Ultimate pH	TB	Ultimate pH	0.882	0.873	0.990
RF	Ultimate pH	SM	Ultimate pH	0.876	0.759	0.866
RF	Ultimate pH	VL	Ultimate pH	0.874	0.871	0.997
AD	Ultimate pH	TB	Ultimate pH	0.869	0.855	0.984
AD	Ultimate pH	PM	Ultimate pH	0.866	0.862	0.996
AD	Ultimate pH	ST	Ultimate pH	0.860	0.855	0.995
SM	Ultimate pH	TB	Ultimate pH	0.839	–	–
AD	Ultimate pH	RF	Ultimate pH	0.828	0.807	0.975
LTL	Ultimate pH	RF	Ultimate pH	0.819	0.789	0.963
AD	Ultimate pH	LTL	Ultimate pH	0.811	0.805	0.992
ST	Ultimate pH	VL	Ultimate pH	0.805	0.748	0.929
SM	Sarcomere length	TB	Shear force	0.795	–	–
PM	Ultimate pH	ST	Ultimate pH	0.792	0.774	0.978
LTL	Ultimate pH	TB	Ultimate pH	0.790	0.766	0.970
LTL	Ultimate pH	PM	Ultimate pH	0.788	0.777	0.986
PM	Ultimate pH	RF	Ultimate pH	0.782	0.773	0.988
SM	Ultimate pH	VL	Ultimate pH	0.778	0.646	0.830
LTL	Ultimate pH	ST	Ultimate pH	0.758	0.756	0.997
AD	Ultimate pH	VL	Ultimate pH	0.746	0.713	0.955
AD	Shear force	LTL	Shear force	0.614	0.566	0.922
SM	Cooking loss	SM	Shear force	0.447	–	–
ST	Shear force	TB	Shear force	0.467	0.221	0.473
RF	Cooking loss	RF	Shear force	0.415	–	–
AD	Ultimate pH	LTL	Shear force	−0.614	–	–
ST	Ultimate pH	VL	Shear force	−0.622	–	–
LTL	Ultimate pH	SM	Sarcomere length	−0.636	–	–
RF	Ultimate pH	TB	Shear force	−0.646	–	–
AD	Ultimate pH	AD	Shear force	−0.661	–	–
PM	Ultimate pH	TB	Shear force	−0.663	–	–
LTL	Ultimate pH	LTL	Shear force	−0.686	–	–
PM	Ultimate pH	SM	Sarcomere length	−0.688	–	–
AD	Ultimate pH	SM	Sarcomere length	−0.693	–	–
AD	Ultimate pH	TB	Shear force	−0.709	–	–
RF	Ultimate pH	SM	Sarcomere length	−0.712	–	–

^1^ Other abbreviations include the following: AD—adductor femoris; LTL—longissimus thoracis et lumborum; PM—psoas major; RF—rectus femoris; SM—semimembranosus; ST—semitendinosus; TB—triceps brachii; VL—vastus lateralis.

**Table 3:** The loading coefficients from the principal component analysis of the quality traits of eight muscles from Australian Rangeland goats. Bold values represent the most influential loadings (>0.25) within each principal component. The difference and proportion of the variance explained by each principal component are also included ^1^.

Quality Trait	Muscle	Principal Component							
		1	2	3	4	5	6	7	8
Cooking loss	AD	−0.11	**0.32**	0.04	0.14	0.02	0.09	−0.20	0.09
	LTL	−0.08	**0.28**	0.01	−0.08	**0.30**	0.07	**0.31**	−0.12
	PM	0.07	**0.26**	0.19	0.17	−0.19	**0.30**	−0.16	−0.16
	RF	−0.01	**0.34**	−0.01	0.18	−0.12	−0.07	−0.08	0.03
	SM	−0.02	**0.39**	−0.04	0.08	−0.03	−0.05	−0.15	−0.06
	ST	−0.05	**0.36**	0.02	0.02	0.03	0.04	0.23	0.15
	TB	0.05	0.03	−0.13	−0.07	**−0.36**	**0.33**	−0.20	**0.55**
	VL	−0.06	0.20	−0.03	−0.19	**−0.38**	**−0.33**	0.11	**−0.29**
Sarcomere length	AD	0.01	−0.02	**−0.38**	0.17	−0.11	0.20	**0.26**	0.10
	LTL	−0.14	−0.14	0.16	−0.07	−0.02	0.16	**0.41**	0.21
	PM	0.17	−0.18	0.07	**0.28**	−0.03	0.18	0.19	−0.03
	RF	−0.10	−0.07	−0.13	−0.04	**0.52**	0.22	**−0.35**	0.03
	SM	**−0.26**	−0.09	0.07	−0.29	0.02	−0.02	0.12	−0.04
	ST	−0.02	−0.16	**0.35**	0.18	−0.10	0.15	0.20	−0.09
	TB	−0.14	−0.11	−0.12	**0.39**	0.21	**−0.27**	0.08	0.12
	VL	0.04	−0.06	**−0.27**	**0.35**	0.16	−0.08	0.01	−0.23
Shear force	AD	−0.20	−0.09	**0.29**	−0.02	0.10	**0.30**	−0.18	−0.06
	LTL	−0.19	0.20	0.21	0.10	0.16	0.02	−0.01	−0.04
	PM	−0.16	**−0.25**	0.09	−0.08	−0.07	−0.22	−0.07	0.18
	RF	−0.19	0.14	0.23	0.05	−0.01	0.07	0.19	0.10
	SM	−0.08	0.01	**0.32**	**0.36**	−0.02	−0.07	0.07	0.11
	ST	−0.11	−0.03	0.29	0.11	0.04	**−0.41**	−0.22	0.18
	TB	−0.23	0.00	0.13	**−0.30**	0.05	0.13	−0.11	−0.21
	VL	−0.12	−0.21	0.02	**0.31**	**−0.25**	0.06	−0.22	−0.23
Ultimate pH	AD	**0.30**	−0.02	0.07	−0.01	0.10	0.04	0.08	−0.12
	LTL	**0.25**	−0.10	0.14	−0.06	−0.18	−0.05	−0.21	−0.15
	PM	**0.28**	0.06	0.06	−0.07	0.06	−0.07	0.04	0.00
	RF	**0.28**	0.04	0.14	−0.01	0.03	−0.02	0.05	−0.06
	SM	**0.27**	−0.04	0.18	−0.01	0.05	0.14	0.07	−0.19
	ST	**0.28**	0.02	0.10	−0.04	0.18	0.06	−0.06	0.02
	TB	**0.29**	0.05	0.08	0.00	0.12	−0.05	−0.07	**0.31**
	VL	0.24	0.05	0.23	−0.08	0.11	−0.23	0.03	0.22
Difference		4.27	1.74	1.01	1.07	0.14	0.14	0.37	0.20
Variance explained, %		30.5	17.2	11.8	8.6	5.3	4.8	4.4	3.3

^1^ Other abbreviations include the following: AD—adductor femoris; LTL—longissimus thoracis et lumborum; PM—psoas major; RF—rectus femoris; SM—semimembranosus; ST—semitendinosus; TB—triceps brachii; VL—vastus lateralis.

## Data Availability

The original contributions presented in this study are included in the article. Further inquiries can be directed to the corresponding author.
